# Transient Global Amnesia and Neurological Events: The Framingham Heart Study

**DOI:** 10.3389/fneur.2013.00047

**Published:** 2013-05-14

**Authors:** José Rafael Romero, Melissa Mercado, Alexa S. Beiser, Aleksandra Pikula, Sudha Seshadri, Margaret Kelly-Hayes, Philip A. Wolf, Carlos S. Kase

**Affiliations:** ^1^Department of Neurology, Boston University Medical CenterBoston MA, USA; ^2^Framingham Heart Study, The National Heart, Lung, and Blood InstituteFramingham, MA, USA; ^3^Department of Biostatistics, Boston University School of Public HealthBoston, MA, USA

**Keywords:** transient global amnesia, cerebrovascular disease, stroke, TIA, Brain MRI

## Abstract

**Background/objective:** Transient global amnesia (TGA) is a temporary amnestic syndrome characterized by lack of other focal neurological deficits. Cerebrovascular disease, migraine and seizures have been suggested as underlying mechanisms. TGA may be a risk factor for cerebrovascular or other neurological events. We studied the relation of TGA, vascular risk factors, brain magnetic resonance imaging (MRI) indices of subclinical ischemia and neurological events in a community-based sample.

**Design/setting:** A total of 12 TGA cases were ascertained using standard criteria by experienced neurologists, and matched to 41 stroke- and seizure-free controls. Vascular risk factors, brain MRI findings, and subsequent cerebrovascular or seizure events were compared in cases and controls.

**Participants:** Framingham Heart Study (FHS) original and offspring cohort participants were included.

**Results:** No significant differences between the groups were observed in the prevalence of vascular risk factors, or brain MRI measures. Few incident stroke/transient ischemic attacks (TIA) (one event among the cases and four in controls) or subsequent seizures occurred in either group. Head CT during the acute event (*n* = 11) and brain MRI (*n* = 7) were negative for acute abnormalities. Electroencephalograms (EEG) (*n* = 5) were negative for epileptiform activity. Extracranial vascular studies were negative for significant stenosis in all cases.

**Conclusion:** In our community-based study TGA was not related to traditional vascular risk factors, or cerebrovascular disease. However, our study is limited by small sample size and power, and larger studies are required to exclude an association.

## Introduction

Transient Global Amnesia (TGA) is a temporary anterograde amnestic syndrome characterized by the lack of other focal neurological signs or symptoms (Hodges and Warlow, 1990a). Patients appear confused, repeatedly asking orienting questions, but with sufficiently intact semantic, working, and procedural memory to be able to continue with everyday activities such as driving a car (Logan and Sherman, 1983). The leading hypotheses to explain the pathophysiology of TGA involve epileptic causes, migraine, and cerebrovascular disease (i.e., ischemia, venous insufficiency). Although TGA is an uncommon disorder with mostly a benign course, (Pantoni et al., 2005) the risk of subsequent neurological events is not entirely clear.

We hypothesized that TGA is related to ischemic cerebrovascular disease and may represent a risk factor for subsequent TIA and stroke. We studied this hypothesis by evaluating the association of TGA with traditional stroke risk factors, measures of subclinical brain ischemic injury on brain MRI, and subsequent stroke, TIA, and seizures in cases from a community-based sample.

## Materials and Methods

The Framingham Heart Study (FHS) is an ongoing longitudinal cohort study that began in 1948 and originally included 5,209 men and women to comprise the Original Cohort, and subsequently enrolled two additional generations. The present sample also included participants of the Offspring Cohort, which enrolled 5,124 participants in 1971.

Transient global amnesia cases were ascertained using standard criteria (Hodges and Warlow, 1990a) by a panel of neurologists. Diagnostic criteria for TGA definition included: (1) acute onset of anterograde amnesia with or without a mild retrograde amnesia, (2) event witnessed, (3) no focal neurological deficits, (4) no alteration of consciousness, (5) resolution of symptoms within 24 h, (6) no loss of self identity, (7) no recent trauma or seizure, (8) no cognitive impairments other than amnesia, and (9) other causes of amnesia excluded.

Controls were matched by FHS cohort, sex, year of birth, and attendance at the most recent FHS exam prior to the TGA episode. Participants in the control group were seizure- and stroke-free at the match time, which was set as the date the TGA event occurred in the cases. A total of 12 TGA cases and 41 controls were included in the study.

Magnetic resonance imaging scans were processed and analyzed as previously reported, (DeCarli et al., 1992) by a reader blinded to the cases and controls’ demographic and clinical information. The presence and location of covert brain infarcts (CBI) and quantitative analysis of white matter hyperintensity (WMH) volume, total cerebral brain (TCBV), and hippocampal (HV) volumes were performed.

Vascular risk factors determined at the examination cycle closest to the TGA event included age, systolic blood pressure, hypertension, diabetes, current smoking, hyperlipidemia.

Cases and controls were compared in terms of: (1) vascular risk factors, (2) brain MRI findings (WMH volume, CBI, TCBV, and HV), and (3) rates of subsequent cerebrovascular events (stroke and TIA) and other neurological events (seizure, migraine). We used conditional logistic regression with case status as outcome to compare the prevalence of cardiovascular risk factors and subclinical brain measures (CBI, WMH, TCBV, and HV) between cases and controls. The comparison of incident TIA, stroke, and other neurological events among cases and controls is only descriptive due to the small number of events.

## Results

During the study period, 12 participants met criteria for a TGA episode. Of these, 10 participants were matched with 4 controls each; one participant had 1 matched control and 1 had none (for a total number of 41 controls). Baseline characteristics of cases and controls showed a mean age of 71 ± 9 years, and 70% were men. None of the cases or controls had history of seizures, migraine, or stroke. The mean duration of TGA events was 2.5 h, ranging from 20 to 300 min. Triggers were observed in five cases, including sexual intercourse, exposure to cold water, and emotional stressors. Mean age for the cases was 71 years, with range of 59–90 years, consisting of 9 men and 2 women.

There was no significant difference with regards to vascular risk factors between the TGA cases and the control group including SBP, hyperlipidemia, and BMI (Table 1). We also did not observe any significant differences between the cases and controls in terms of the brain MRI measures studied (CBI, WMH, TCBV, HV) (Table 1). There were too few participants with diabetes, smoking, or covert infarcts for statistical comparison.

**Table 1:** **Baseline clinical and MRI characteristics in entire sample and comparison between cases (*N* = 12) and controls (*N* = 41)**.

Clinical characteristics	Entire sample (*N* = 53)	Odds ratio (CI)	*p*-Value
Age (years, mean ± SD)	71 ± 9	–	–
Males (%)	70%	–	–
Hypertension (*n*, %)	21 (70%)	0.82 (0.11–5.81)	0.838
Systolic blood pressure (mmHg, mean ± SD)	131 ± 16	1.13 (0.58–2.22)	0.717
Diabetes mellitus (*n*, %)	2 (9%)	–	–
Smokers (*n*, %)	2 (4%)	–	–
Total cholesterol level (mg/dl, mean ± SD)	186 ± 34	1.00 (0.42–2.39)	0.997
HDL cholesterol level (mg/dl, mean ± SD)	52 ± 16	1.55 (0.72–3.30)	0.262
Body mass index (mean ± SD)	29 ± 5	1.08 (0.51–2.26)	0.846
**MRI characteristics**	**(*N* = 27)**		
White matter hyperintensity volume	0.09 ± 0.22	0.22 (0.04–1.33)	0.098
Total brain volume	78 ± 3	2.48 (0.49–12.47)	0.271
Hippocampal volume	0.33 ± 0.06	0.53 (0.16–1.78)	0.303
Covert brain infarcts	3 (11%)	–	–

Multiple logistic regression analysis. CI, Confidence Interval. *Too few participants with diabetes, smoking, or covert infarcts for statistical comparison.

All of the matched TGA cases underwent non-contrast CT imaging at the time of the event and seven had MRIs performed. The majority of the cases had head CT performed within 1 day of the event (mean = 2.5 ± 7.8 days). Head CT was normal in 8 of the 11 participants, and showed only diffuse atrophy in 3 of them. Brain MRI, performed within an average of 12.3 ± 21 days, was normal in three of the seven participants, showed old lacunar infarcts in one participant, and diffuse atrophy in four participants. None of the participant’s MRIs reported evidence of acute infarction. EEG was performed in five participants within an average of 2.3 ± 3.2 days. The test was normal in three participants, and showed focal slowing in two of them (one bitemporal, one left temporal), but none showed evidence of epileptiform activity.

Cases and controls were followed for a mean period of 5.5 years (range 2–13). Four incident stroke/TIA events occurred in the control group and one event occurred among the TGA cases. Further statistical analyses were limited due to small number of events and low power.

## Discussion

Evaluation of our TGA cases during the acute event was non-revealing, and no definite evidence of acute stroke was found in those who underwent CT and MRI, or seizures in those with EEG testing. Although prior studies have found evidence of hypoperfusion in the mesial temporal lobes supporting the hypothesis of a hypoxic-ischemic insult as the cause of TGA, (Hodges, 1998; Di Filippo and Calabresi, 2007) we did not observe brain MRI findings in our study to suggest higher prevalence of subclinical ischemic brain injury or hippocampal atrophy in TGA cases.

In prior studies, the incidence of subsequent stroke or TIA has been noted to be significantly higher in TIA patients than in TGA subjects (15.4 vs. 0% in one study and 30 vs. 5% in another) (Hodges and Warlow, 1990b; Zorzon et al., 1995). Although we observed subsequent events in our sample, the number of events was too small to allow for statistical comparison. Furthermore, we also did not find a different vascular risk factor profile in patients with TGA compared to controls.

Epileptic causes have also been suggested as underlying mechanisms for TGA. Certain anatomical and physiological characteristics of the hippocampi may predispose it to partial seizures that could present as transient amnestic episodes, (Chang and Lowenstein, 2003) and previous reports have attributed TGA to epilepsy in 4.5% of the cases studied. (Zorzon et al., 1995) We did not find evidence to support epilepsy as the etiology of TGA events in our cases as their EEG studies were unrevealing and none of them had subsequent seizures. EEG was not done during the acute TGA events and was only performed on half of the cases, thus we cannot exclude seizure as a potential mechanism.

Migraine also has been suggested as a possible mechanism since previous studies have found a higher prevalence of migraine headache in TGA cases (23%) as compared to TIA (9%) and normal controls (6%) (Hodges and Warlow, 1990b; Zorzon et al., 1995). A possible explanation for migraine as an etiology for TGA may be transient spreading depression of the bilateral hippocampi (Evans and Lewis, 2005). In our study none of the cases had history of migraine or subsequent migraine following the TGA events.

## Concluding Remarks

In our community-based study we did not find a relation of TGA with traditional vascular risk factors, or subclinical cerebrovascular disease. However, our study is limited by small sample size and power, and larger studies are required to exclude an association with subclinical cerebrovascular disease or with subsequent clinical events.

## Conflict of Interest Statement

The authors declare that the research was conducted in the absence of any commercial or financial relationships that could be construed as a potential conflict of interest.
